# Posterior Reversible Encephalopathy Syndrome in a Patient with Septic Shock: A Case Report

**DOI:** 10.5811/cpcem.1461

**Published:** 2023-06-25

**Authors:** Eric Boccio, Fiore Mastroianni, Todd Slesinger

**Affiliations:** * UMass Chan Medical School – Baystate, Department of Emergency Medicine, Department of Healthcare Delivery & Population Sciences, Springfield, Massachusetts; † New York University Long Island School of Medicine, Department of Medicine, Mineola, New York; ‡ Herbert Wertheim College of Medicine, Florida International University, Department of Emergency Medicine, Miami, Florida

**Keywords:** posterior reversible encephalopathy syndrome, PRES, septic shock, case report

## Abstract

**Introduction:**

Posterior reversible encephalopathy syndrome (PRES) is a reversible condition with nonspecific neurologic and characteristic radiologic findings. Clinical presentation may include headache, nausea, vomiting, altered mental status, seizures, and vision changes. Diagnosis is confirmed through T2-weighted brain magnetic resonance imaging (MRI) showing bilateral hyperintensities in the white matter of posterior circulatory regions.

**Case Report:**

We report a case of PRES in a patient suffering from complicated diverticulitis. Following medical management in the emergency department, the patient deteriorated, becoming hypotensive and altered. Bowel resection under general anesthesia was performed. Postoperative brain MRI demonstrated bilateral and symmetric T2 signal hyperintensities suggestive of PRES. Following supportive treatment, the patient was discharged from the surgical intensive care unit on postoperative day 21 with no residual deficits.

**Conclusion:**

It is important to recognize the nonspecific neurologic symptoms associated with PRES. Emergency physicians should suspect acute PRES when managing patients with prolonged or unexplained encephalopathy, while recognizing that hypertension need not be present.

## INTRODUCTION

Posterior reversible encephalopathy syndrome (PRES) (known also as posterior reversible leukoencephalopathy and reversible posterior cerebral edema syndrome) is both a clinical and radiological entity, since it is defined by nonspecific neurologic symptoms and characteristic radiologic findings. Clinical presentation of PRES may include headache, nausea, vomiting, altered mental status, seizures, and visual loss or disturbance. Initial findings may not be specific enough to effectively raise clinical suspicion or confirm diagnosis. In contrast, neuroimaging demonstrates characteristic transient changes, and diagnosis is typically made based upon T2-weighted magnetic resonance imaging (MRI) findings of hyperintensities predominantly in the posterior white matter, and less so in the surrounding cerebral cortex.

Although the cause of PRES is not clearly established, dysfunction in cerebral autoregulation and degradation of the blood-brain barrier has been posited.^1^ Posterior reversible encephalopathy syndrome is a mostly reversible condition, and treatment should focus on alleviating the suspected underlying cause. In rare instances, symptoms of PRES are not reversible, and patients may suffer from cerebellar herniation and residual neurologic deficits. Many reports have described PRES in the setting of uncontrolled hypertension secondary to pre-eclampsia and eclampsia, immunosuppressive therapies, renal insufficiency, post-transplantation, and lupus,^2^–^5^ in contrast-related anaphylaxis and alcohol withdrawal,^6^ as well as in the postoperative setting following spinal surgery^7^ and thoracotomy.^8^ Less frequently reported are cases of PRES in the setting of hypotension and various shock states. The etiology of this hypotensive subset of patients may still be due to dysfunction of cerebral blood flow; however, hypertension as a primary mechanism is nonexplanatory.

This case report illustrates a novel presentation of PRES diagnosed in a patient suffering from septic shock secondary to complicated diverticulitis who required vasopressors and surgical bowel resection under general anesthesia.

## CASE REPORT

A 68-year-old female with a known history of diverticulosis, hypercholesterolemia, and hypothyroidism presented to our emergency department (ED) complaining of three days of worsening abdominal pain. The pain was severe in the lower quadrants and more so in the left lower quadrant. Review of systems was notable for fever, chills, and diarrhea. She denied any associated nausea or vomiting. In a past exacerbation of her diverticulosis she had experienced lower gastrointestinal bleeding. Her last colonoscopy had been performed three years prior to date of presentation and showed diverticula. Current medications included aspirin, levothyroxine, atorvastatin, and ramipril. On physical exam, the abdomen was diffusely tender and distended with guarding. There were no other pertinent findings. Initial ED vital signs were within normal limits, with the exception of an elevated oral temperature that ranged between 99.0–102.9° Fahrenheit throughout the ED course.

Computed tomography (CT) of the abdomen and pelvis with iodinated intravenous contrast demonstrated a perforated diverticulum in the sigmoid colon and showed local extraluminal air. On admission, laboratory results revealed mild leukocytosis (11,500/microliters [uL]) (reference range 4,000–11,000/uL) with increased neutrophils (9,400/uL) (2,500–6,000/uL). There were also trace leukocyte esterase and 5–10 white blood cells per high-power field found in urinalysis. The patient was admitted to the surgical service for observation and initially treated with piperacillin/tazobactam.

While inpatient, the patient’s clinical condition deteriorated resulting in fluid-refractory tachycardia and hypotension. She was intubated and taken to the operating room for surgical resection of the perforated bowel under general anesthesia, and a successful Hartman procedure was performed with minimal blood loss. Postoperatively, the patient was maintained on norepinephrine for hemodynamic support as well as fentanyl, midazolam, and propofol for post-intubation sedation. During her postoperative recovery, the patient failed to return to her baseline normal mental status, becoming increasingly agitated, delirious, and uncooperative. Soft physical restraints were maintained on both wrists.

**CPC-EM Capsule**

What do we already know about this clinical entity?

*Posterior reversible encephalopathy syndrome (PRES) has nonspecific neurologic and characteristic radiologic findings typically described in the setting of hypertension.*

What makes this presentation of disease reportable?

*We present an unusual case of PRES in a patient suffering from septic shock secondary to complicated diverticulitis.*

What is the major learning point?

*Posterior reversible encephalopathy syndromeshould be considered in the differential of prolonged or otherwise unexplained encephalopathy, even in normo- and hypotensive patients.*

How might this improve emergency medicine practice?

*Awareness of PRES should facilitate ordering of appropriate neuroimaging, specifically brain magentic resonance imagaing, allowing for confirmation of characteristic radiologic findings.*

**Image 1:**
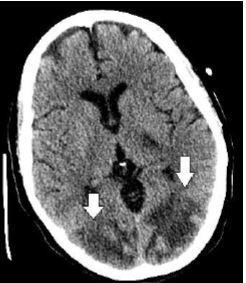
Computed tomography head imaging performed on postoperative day seven demonstrating multiple hypodensities in white matter of posterior parietal and occipital lobes (arrows).

A CT head without contrast was performed on postoperative day 7 to investigate persistent mental status changes and visual deficits. Multiple hypodensities in the white matter of the posterior parietal and occipital lobes were identified (Image 1).

**Image 2:**
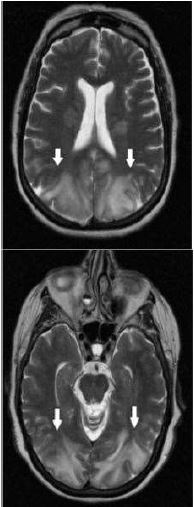
Magnetic resonance imaging brain obtained on postoperative day eight demonstrating T2-weighted hyperintensities (arrows) localized to white matter of bilateral posterior cerebral cortex (bottom) and cerebellum (top).

The next day, MRI brain without contrast demonstrated bilateral and symmetric T2-weighted signal hyperintensities confined to the white matter of the posterior cerebral cortex and cerebellum (Image 2).

The symmetrical nature of the abnormality and its confinement to the posterior cortex made it highly suspicious for PRES. Following the MRI, a 24-hour video electroencephalogram study was performed, which showed moderate diffuse cortical dysfunction and background slowing. The patient continued to receive supportive measures over several days and was discharged on postoperative day 21 with no residual neurologic deficits.

## DISCUSSION

Posterior reversible encephalopathy syndrome is referred to as a neurotoxic state characterized by variable clinical signs and symptoms, unique radiologic features, and a general reversibility of condition. Acute PRES may present as severe headache, nausea, vomiting, altered mental status, seizures, stupor, and visual loss or disturbance.^9^ The relative complexity and lack of specificity often make diagnosis difficult based on clinical symptoms alone.

Despite the widely diverse clinical presentation of PRES, its appearance on neuroimaging is better understood. On T2-weighted MRI brain, PRES can be seen as bilateral hyperintensities predominantly in the white matter of the posterior cerebral hemispheres although asymmetrical and partial image expressions are common. On diffusion-weighted imaging modalities, such as T1-weighted MRI brain, PRES is seen as hypointensities or isointensities localized in the same regions.^10^ Affected regions are hypoattenuating on CT head. Because findings on CT are inconsistent, MRI has become the favored, if not essential, imaging modality for the diagnosis of PRES.^11^

Edema is predominantly present in focal regions of the posterior circulation territories, although anterior circulation structures of the brain are also involved: parietal or occipital regions (98%), frontal lobes (68%), inferior temporal lobes (40%), and cerebellar hemispheres (30%).^12^ In the cerebellar hemispheres, three main patterns, and partial or asymmetric expression of these patterns, have been noted: holohemispheric watershed (23%); superior frontal sulcus (27%); and dominant parietal-occipital (22%). Presence of lesions in the basal ganglia, brain stem, and deep white matter of the brain, including the splenium, is also indicative of PRES.^13^ Findings have suggested that there may be a higher incidence of atypical regions of involvement and uncommon imaging manifestations than previously perceived, making the radiologic presentation of PRES slightly more variable.^6^

The cause of acute PRES is not completely understood, and explanations of the mechanism leading to brain edema have been controversial. Controversy involves the role of hypertension and whether the edema is cytotoxic or vasogenic in origin, resulting from hyperperfusion or hypoperfusion. While most cases (70%) have been associated with systemic hypertension, or, more accurately, a rapid rise in blood pressure, a significant portion of patients have documented normal or mildly elevated blood pressures.^14^ Diffusion-weighted MRI of patients with hypertensive PRES suggests that the condition is attributable to hyperperfusion leading to disruption of the blood-brain barrier and resultant vasogenic edema, not ischemia nor infarction.^11^ This theory suggests that the lesions seen in PRES are caused by abnormal autoregulatory functions, which control blood flow in the context of rapid increases in systemic blood pressure. Failure in this mechanism leads to a breakdown of endothelial cells in the cerebral vasculature, and the increased permeability leads to interstitial and vasogenic edema and increased intracranial pressure.^2^

Left untreated, vasogenic edema will progress to cytotoxic edema, ischemia, and infarction of brain tissues. Management strategies should be initiated early and focused on treating the underlying cause while eliminating exacerbating factors. Supportive care and symptom management are paramount. Hydration, electrolyte correction, airway monitoring and protection, and ventilatory support should be considered, especially if the patient is altered, obtunded, or suffering from status epilepticus.^15^ If hypertension is believed to be the main cause, antihypertensives should be administered and blood pressure must be continually monitored.

When cytotoxicity is suspected, the dosage of the offending agent should be lowered, and medication may need to be withdrawn completely. In the case of pre-eclampsia and eclampsia, emergent delivery of the fetus is recommended. Pro-inflammatory states such as sepsis should be managed with antibiotics, hemodynamic management, and corticosteroids. There are currently no clinical trials assessing the efficacy and safety of hyperosmolar therapies in PRES.

## CONCLUSION

We have presented the case of an adult female suffering from septic shock secondary to complicated diverticulitis who developed posterior reversible encephalopathy syndrome. This case highlights the need to recognize the nonspecific neurologic symptoms associated with PRES and encourages emergency physicians to consider the diagnosis when approaching the differential of altered mental status. It is important to note that hypertension need not be present, as a subset of patients with PRES may be normotensive or hypotensive. Sepsis and septic shock are very common presentations in the ED setting, and encephalopathy must be considered a sign of end organ dysfunction.

It is plausible that acute PRES in septic shock patients exhibiting signs of encephalopathy is underdiagnosed due to a lack of clinical suspicion and unawareness of the disease process. For patients with prolonged or otherwise unexplained alteration in mental status, awareness and rapid ordering of appropriate neuroimaging will allow for confirmation of characteristic radiologic findings and prevent misdiagnosis, unnecessary testing, and delays in treatment. Reversible with appropriate treatment, PRES requires early recognition; diagnosis is crucial to prevent the complicating factors associated with prolonged PRES, specifically cytotoxic edema, brain ischemia and infarction, and death.
